# Arteriosclerosis in Brazil. Findings from the Brazilian Longitudinal Study of Adult Health (ELSA-Brasil)

**DOI:** 10.1590/1516-3180.2018.1362280218

**Published:** 2018-01-09

**Authors:** Paulo Andrade Lotufo

**Affiliations:** I MD, DrPH. Full Professor, Department of Internal Medicine, Faculdade de Medicina da Universidade de São Paulo (FMUSP), São Paulo (SP), Brazil.

*Do not panic if you confuse “atherosclerosis” and “arteriosclerosis”. Few people, including teachers and researchers in the medical world, can describe in few words the difference between these pathological conditions, which have distinct impacts on clinical practice and epidemiological studies (Lotufo PA, SPMJ, 2016).*^1^

Briefly, arteriosclerosis relates to increased stiffness of the walls of major arteries and is correlated with greater age and higher blood pressure. On the other hand, atherosclerosis is a pathological process involving the endothelium of the tunica media and intima and relates to oxidation of cholesterol followed by a complex inflammatory chain. The degree of arteriosclerosis can be measured by some of the devices addressing different physiological characteristics of the blood circulation throughout the arterial tree.^2^

The most popular measurement is pulse-wave velocity, most commonly between the carotid and femoral arteries. This measurement is relatively easy and safe to make, but its clinical use is not yet recommended. However, clinical and epidemiological studies are changing the meanings of vascular diseases and hypertension. The physiological index most commonly determined has been the carotid-femoral pulse-wave velocity (cf-PWV). The higher the cf-PWV is, the more rigid and less distensible the major arteries will be. Stiffness of the aorta is associated with adverse health outcomes relating to hemodynamics, such as left-ventricular hypertrophy and diastolic heart failure. Moreover, high cf-PWV increases pulsatility in the capillary tree in organs that have high flow and low resistance, such as the brain (leading to white-matter lesions) and the kidney (leading to low glomerular filtration rate and albuminuria).^3^ An increase of one standard deviation in aortic PWV has been found to be associated with nonfatal cardiovascular events (47%), cardiovascular mortality (47%) and all causes of death (42%).^4^

The Brazilian Longitudinal Study of Adult Health (ELSA-Brasil) is a cohort of 15,105 women and men aged 35-74 years who have been followed up since a baseline visit in 2008-2010 that is evaluating both atherosclerosis and arteriosclerosis. A summary of the findings of atherosclerosis in ELSA-Brasil has been published elsewhere.^1^ Here, we aim to describe the findings relating to arteriosclerosis or, more precisely aortic stiffness as measured through the pulse-wave velocity.^5^^,^^6^

**Figure 1:**
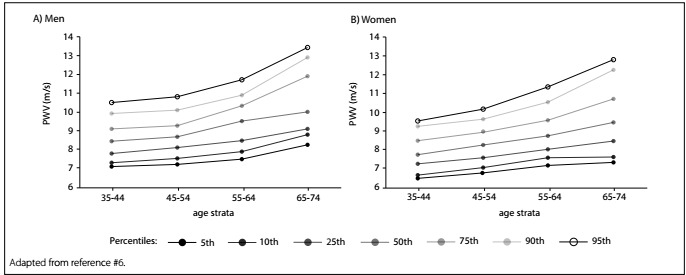
Percentiles of carotid-femoral artery pulse wave velocity according to age strata among apparently healthy participants at the ELSA-Brasil baseline (2008-2010).

Figure 1 shows the cf-PWV according to age among 2158 apparently healthy men and women and indicates that it has a monotonic association with age. Among those subjects, men had higher values than women. There were no differences in the slope of cf-PWV versus age according to ethnicity/skin color after adjustment, thus confirming that age and mean arterial pressure have a substantial effect on pulse-wave velocity.^5^

Other classical risk factors have been seen to slightly influence the slope of the “pulse-wave velocity versus age” curve, such as diabetes, obesity and smoking, but the most influential risk factor is blood pressure. Nonetheless, the impact of these risk factors is marginal compared with the influence of aging, with a mean elevation of 0.05 m/s for each year of age, compared with 0.1 m/s per year for the whole ELSA-Brasil cohort including people with cardiovascular risk factors.^6^

In conclusion, arterial stiffness is strongly associated with aging. However, diminishing the burden of traditional risk factors can moderate the evolution of arteriosclerosis.
